# Supplementary material for: Process of Introduction of Australian Braford Cattle to South America: Configuration of Population Structure and Genetic Diversity Evolution
Source: Animals (Basel). 2022 Jan 23;12(3):275. doi: 10.3390/ani12030275 (PMC8833419; doi:10.3390/ani12030275)
Supplement: Supplementary file 1 [file animals-12-00275-s001.zip › Table S1.pdf]

Table S1. Braford cattle breed data fact sheet.

**Breed standard**	For both sexes, the general conformation of Braford individuals should present good muscular development, mainly in the arm, leg and loin. The color of the coat must present the character ¨pampa¨ demonstrating the contribution of the Hereford breed. Females at 2 years must weigh at least 400kg and males 550kg. The weights for adult animals are as follows: 950 kg, for males and 650 kg for females.
**Criteria for herdbook inscription**	The records for registration of Braford breeders in the ABA are divided into the following sections: Braford of origin (Bo), preparatory, controlled and registered, advanced and final. Braford of origin is defined as general Braford-type bellies that show some combination of Hereford and Zebu blood detectable in their external appearance and that are presented for inspection. To be entered in the Preparatory Register, the offspring from matings between at least one of the original breeds with Braford or those with each other and the following varieties are accepted: Braford ¼, Braford 3/8, Braford ½, Braford 5/8 and Braford ¾. Male and female Braford offspring from registered breeders, both in the Preparatory, Controlled, Registered, Advanced and Definitive registries that come from a collective service without parental identification are accepted to enroll in the Controlled Registry, even if they are 3rd, 4th or more generations. As well as those that come from a collective service with identification of parents in which at least one male does not have DNA analysis; Braford male and female offspring from bulls or bellies registered in the Preparatory registry, even if they had been generated in services with identification of their fathers, and Braford males and females from Bo mothers who had received services of any kind. For the Registered Registry, those animals that come from: an individual service, identified mother and father, must be one of these as a minimum of controlled registry; a collective service, individualized mother and with individualized parents, these must be at least a controlled record. For the Advanced Registration, accepted male and female offspring that come from individualized parents as a minimum of Registered registration are incorporated. For the Definitive registration, accepted male and female offspring that come from individualized parents at least of Advanced registration are incorporated.
**Extension**	Perfectly adapted to extensive systems with stational supplementation.
**Herds size**	The average herd size is 643.10 animals per herd. The total population is 115,757 animals distributed in 180 herds.
**Orientation**	Braford calves have an outstanding butcher conformation, have good growth capacity, vigor, health and resistance to adverse conditions, and excellent behavior in the different rearing and finishing systems, for which they are appreciated and valued by the consumer industry and export. Braford steers show great hook performance, and their half cattle show very good conformation and degrees of finishing.
**Husbandry and health practices**	The rusticity of the breed allows these animals to be raised in an extensive regime, taking advantage of the resources of the pastures and with the complement provided in times of scarcity. Practices strongly influenced by the location where the animals are produced.
**Reproductive management**	Most of the services are performed by natural mating, with approximately 15% being through the use of AI. Braford 3/8 bulls reach puberty at 13.1 months of age, with a body weight of 333 kg and a scrotal circumference of 27.5 cm. While the females have their first service depending on the diet, those fed on cultivated pastures enter at 15 months with approximately 420 kg, those fed on natural fields, enter between 18 and 24 months with more than 280 kg of live weight.
**Main products**	The steers fit into all marketing categories, achieving an animal with rapid conversion of grass into meat, high precocity and easy finishing in field with 440 to 460 kg at 22 months of age.
**Official recognition**	In 1969, the International Braford Association (IBA) was founded to begin registering animals of the Braford breed.
**Protection structures status**	In 1962 the Australian Braford Society was created. In Latin America, the breed began to be used in the 1970s; In 1973 the Braford and Cebu del Uruguay Breeders Society was founded, in 1984 the Braford Argentina Association and in 1990 the Paraguayan Association of Braford breeders were created.

For more information, access the following links: https://www.braford.org.ar/, http://www.braford.org.au, http://www.braford.com.py, http://www.braford.com.uy.
