# Supplementary material for: Process of Introduction of Australian Braford Cattle to South America: Configuration of Population Structure and Genetic Diversity Evolution
Source: Animals (Basel). 2022 Jan 23;12(3):275. doi: 10.3390/ani12030275 (PMC8833419; doi:10.3390/ani12030275)
Supplement: Supplementary file 1 [file animals-12-00275-s001.zip › Table S2.pdf]

Table S2. Descriptive statistics of average age (years) of the parents at the birth of their offspring and generation intervals or generation length (years) for the four gametic routes in the Braford cattle breed.

Average age (years) of the parents at the birth of their offspring						
Parameter / Population set	Mean age of the parents at the birth of their offspring	Bull to son	Cow to son	Bull to daughter	Cow to daughter	Total
Historical (n=36678)	N	85085	84656	121368	122216	413325
	Mean	8.45	7.76	20.83	20.21	15.42
	SD	7.24	8.04	12.78	13.15	12.67
	SEM	0.02	0.03	0.04	0.04	0.02
Current (n=13968)	N	37839	35015	53724	49830	176408
	Mean	6.66	6.50	15.26	13.72	11.24
	SD	4.55	3.97	14.03	13.55	11.62
	SEM	0.02	0.02	0.07	0.07	0.03
Generation intervals, generation length or average age (years) of the parents at birth of their offspring that in their turn will produce the next generation of breeding animals						
Parameter / Population set	Gametic route	Bull to son	Cow to son	Bull to daughter	Cow to daughter	Total
Historical (n=36678)	N	1982	23031	1916	29898	56827
	Mean	5.92	6.51	15.82	20.05	13.93
	SD	7.13	10.07	13.24	13.61	13.80
	SEM	0.16	0.07	0.30	0.08	0.06
Current (n=13968)	N	370	6288	370	8569	15597
	Mean	6.38	6.69	14.68	15.63	11.78
	SD	4.19	4.61	14.03	14.48	12.18
	SEM	0.22	0.24	0.73	0.75	0.10
